# Keep Calm and Carry On: The Relations Between Narrative Coherence, Trauma, Social Support, Psychological Well-Being, and Cortisol Responses

**DOI:** 10.3389/fpsyg.2021.558044

**Published:** 2021-02-11

**Authors:** Lauranne Vanaken, Tom Smeets, Patricia Bijttebier, Dirk Hermans

**Affiliations:** ^1^Centre for the Psychology of Learning and Experimental Psychopathology, Faculty of Psychology and Educational Sciences, KU Leuven, Leuven, Belgium; ^2^Department of Medical and Clinical Psychology, Tilburg School of Social and Behavioral Sciences, Tilburg University, Tilburg, Netherlands; ^3^Faculty of Psychology and Educational Sciences, School Psychology and Development in Context, KU Leuven, Leuven, Belgium

**Keywords:** narrative coherence, autobiographical memory, trauma, social support, psychological well-being, state anxiety, stress, cortisol

## Abstract

In order to explain trauma resilience, previous research has been investigating possible risk and protective factors, both on an individual and a contextual level. In this experimental study, we examined narrative coherence and social support in relation to trauma resilience. Participants were asked to write about a turning point memory, after which they did the Maastricht Acute Stress Test, our lab analog of a traumatic event. Following, half of the participants received social support, whereas the other half did not. Afterwards, all participants wrote a narrative on the traumatic event. Moment-to-moment fluctuations in psychological and physiological well-being throughout the experiment were investigated with state anxiety questionnaires and cortisol measures. Results showed that narratives of traumatic experiences were less coherent than narratives of turning point memories. However, contrary to our predictions, coherence, and, in particular, thematic coherence, related positively to anxiety levels. Possibly, particular types of thematic coherence are a non-adaptive form of coping, which reflect unfinished attempts at meaning-making and are more similar to continuous rumination than to arriving at a resolution. Furthermore, coherence at baseline could not buffer against the impact of trauma on anxiety levels in this study. Contrary to our hypotheses, social support did not have the intended beneficial effects on coherence, neither on well-being. Multiple explanations as to why our support manipulation remained ineffective are suggested. Remarkably, lower cortisol levels at baseline and after writing about the turning point memory predicted higher coherence in the trauma narratives. This may suggest that the ability to remain calm in difficult situations does relate to the ability to cope adaptively with future difficult experiences. Clinical and social implications of the present findings are discussed, and future research recommendations on the relations between narrative coherence, social support, and trauma resilience are addressed.

## Introduction

Up to 69% of individuals in the general population report having been exposed to a traumatic event over the course of their life (Resnick et al., 1993). However, the estimated prevalence of Post-Traumatic Stress Disorder (PTSD) is suggested to be much lower, about 8%, as reported in the National Comorbidity Survey (Kessler et al., 1995). These results indicate that it is crucial to investigate why some people are able to cope better with hardship than others (Perkonigg et al., 2000). Previous research has examined possible risk and protective factors that can help explain trauma resilience, both on an individual and a contextual level, like prior psychological adjustment, peritraumatic emotional responses, and posttraumatic social support (Ozer et al., 2003; Charney, 2004; Southwick et al., 2014; Sippel et al., 2015).

In terms of individual factors, a large number of studies have focused on the trauma memory itself and more specifically on the narrative structure of traumatic life experiences (Crespo and Fernández-Lansac, 2016). Research on the coherence of traumatic events suggests that trauma narratives are generally poorer in structure as compared with narratives about positive events (Brewin et al., 1996; Brewin, 2001; Tuval-Mashiach et al., 2004). This line of research stems from Horowitz' (1976) theory of stress response syndromes, outlining how initial incomplete processing of the traumatic experience together with defense mechanisms like avoidance can cause the memory of the traumatic event to remain incoherent and incomplete. Evidence for this theory is quite wide-ranged; for instance, in a study of Filkuková et al. (2016), terrorist-attack survivors who showed high levels of PTSD provided trauma narratives that contained less-organized thoughts in combination with less-detailed descriptions of actions and dialogs, indicative for a lower quality of recall and lack of trauma processing. However, another line of research adheres to the theory that traumatic events are considered to be central in the life story and are hence not more fragmented or incoherent than happy memories but often rather remembered more vividly and with more detail (Berntsen and Rubin, 2006; Rubin et al., 2008; Rubin, 2011). For instance, Rubin (2011) used multiple measures to assess coherence of trauma narratives, in comparison with other memory types, of PTSD patients and healthy controls. He did not find any differences in any of the coherence measures, in narratives of traumatic events in comparison with positive or important events, or for patients suffering from PTSD. In sum, there has been extensive debate on the coherence of traumatic narratives in the literature (e.g., Rubin et al., 2016). Hence, in a first research question, we wanted to compare within individuals their memory coherence of a non-traumatic important event, to their memory coherence of an experimentally induced traumatic event. This gives the advantages of having a reference measurement before the trauma took place. Furthermore, it creates the benefit having an experimental trauma that is kept constant between individuals, reducing interindividual differences in traumatic experiences and hence limiting the possible effects of variety in trauma topics on the coherence of trauma narratives.

As briefly touched upon earlier, the reason why narratives are investigated in terms of their narrative structure is because coherence seems to reflect the individual's abilities to cope with the event and hence can indicate trauma resilience (Horowitz, 1976; Fivush and Baker-Ward, 2005; Southwick et al., 2014; Vanderveren et al., 2020a). For instance, in terror-attack survivors (Tuval-Mashiach et al., 2004) and individuals who recently got divorced (Kellas and Manusov, 2003), narrative coherence was associated to better mental health. The relations between narrative coherence and psychological well-being are also found in the expressive writing literature (Pennebaker and Beall, 1986; Pennebaker, 2000). It has been suggested that over the course of the 4-day writing protocol, the increase in use of causal and insight words is associated to meaning-making, better psychological health, and better physical health (Klein and Boals, 2010; Boals et al., 2011). In other words, the extent to which the trauma narrative is coherently shaped, seems to reflect how people come to cope with the traumatic event and can forecast the process of meaning-making and recovery (Brewin, 2003; Booker et al., 2020). However, there are some empirical inconsistencies in the association between narrative coherence and mental health, including observations in the opposite of direction of what is usually hypothesized (e.g., Stadelmann, 2006; Chen et al., 2012). Sometimes, it has been observed that higher levels of coherence can be associated with more depressive symptoms, higher levels of rumination, or more symptoms of PTSD (Sales et al., 2013; Waters et al., 2013; Vanderveren et al., 2020b). Hence, our second research question concerns the association between the coherence of traumatic events and psychological well-being. A critical evaluation of the literature on the relation between coherence and mental health suggests that the majority of evidence is still in favor of more coherence being associated with higher levels of well-being (e.g., Adler et al., 2016). Therefore, we predict that coherent narratives reflect higher well-being, in this study operationalized as lower state anxiety, in comparison with incoherent narratives. We chose to work with state anxiety measures since they are not only able to capture momentary fluctuations but are also a particularly relevant measure in order to measure well-being after trauma, since anxiety disorders form the main risk to our psychological health after trauma exposure (Ayazi et al., 2014).

Moreover, coherence is not only investigated as a phenomenological characteristic of trauma narratives that reflects adjustment after trauma exposure. It has also been examined as an individual difference variable of non-traumatic autobiographical memories, as a buffering factor against traumatic experiences before they even take place (Vanaken et al., 2020; Vanderveren et al., 2020a). Interestingly, in preschool children, higher coherence in story-stem narratives buffered against the impact of maternal stress to prevent development of internalizing symptoms (Stadelmann et al., 2015). In a study of Mason et al. (2019), the ability of mothers to form an integrated narrative of their parenting experiences could forecast their psychological and biological trauma resilience, in terms of lower parenting stress and less telomere shortening. Similarly, in a recent study of Vanderveren et al. (2020a), baseline narrative coherence could predict future coping with the stressful experience of failing on exams. Students with a higher baseline narrative coherence reported less subjective distress and less impact of the failing on exams, in comparison with students with low baseline narrative coherence. Furthermore, the highly coherent students ruminated less and indicated fewer negative changes in their meaning of life after failing their exams. Relatedly, in our own recent work (Vanaken et al., 2020), higher baseline narrative coherence, particularly of narratives about significant positive autobiographical experiences, predicted higher levels of psychological well-being and fewer depressive symptoms, 2 years later during the COVID-19 pandemic. However, besides these studies, to our knowledge, not much research has been done on the buffering effects of narrative coherence against the development of psychopathology after trauma exposure. Consequently, our *third* research question involves the investigation of the buffering effect of coherence of a non-traumatic narrative against the impact of a traumatic event on psychological well-being. We hypothesize that higher narrative coherence before being exposed to trauma can predict more adaptive coping skills and hence a smaller decrease in psychological well-being after trauma exposure.

As indicated earlier, not only individual factors but also contextual factors like posttrauma social support have been topic of investigation to help explain trauma resilience (Ozer et al., 2003, Sippel et al., 2015). Traumatic experiences and consequent narrative formation do not occur solely inside our heads, they take place in a social context (Bavelas et al., 2000; Alea and Bluck, 2003; Ajdukovic et al., 2013; Sijbrandij and Olff, 2016). For instance, Clark (1994) argued that “narratives seem different from conversations, because they seem to be produced by individuals speaking on their own (…) But appearances belie reality. Narratives rely just as heavily on coordination among the participants as conversations do. It is simply that the coordination is hidden from view” (Clark, 1994, pp. 1006–1007). Humans are wired to share personal experiences with others, since it adds to the development and maintenance of social relationships, which is line with the social function of autobiographical memory (Rimé et al., 1998; Bluck and Alea, 2002; Pasupathi and Carstensen, 2003). This social sharing of experiences is not a one-way street; the process of coming to a narrative is a joint activity occurring in a social context, called co-construction (Pasupathi, 2001). Several researchers have indeed investigated the impact of the social context on our memories (Pasupathi et al., 1998), the way we talk about them (Grysman and Mansfield, 2017), and how we feel after talking about them (Nils and Rimé, 2012). For instance, Pasupathi and Rich (2005) found that inattentive listeners have an impairing effect on self-perception in personal narratives. Bavelas et al. (2000) also found that listeners' contributions are essential in face-to-face dialog. However, no research has been done on the effect of social support on narrative coherence in particular. Hence, in a *fourth* question, we will investigate the impact of social support on narrative coherence, hypothesizing that support will aid the listener in constructing a coherent account of the experienced events.

Furthermore, not only are narrative processes inevitably impacted by our social environment, so is our psychological well-being. There is a large amount of evidence on the relations between social support and both mental and physical health (Ozbay et al., 2007; Harandi et al., 2017) and on the benefits of posttraumatic social support (Ozer et al., 2003; Sippel et al., 2015; Liu et al., 2017). In these studies, social support comes out as the number one contextual factor that can explain variability in PTSD symptoms between individuals. Experiencing a sense of belongingness turns out to be one of human's primary needs (Maslow, 1967; Baumeister and Leary, 1995) and hence has a crucial impact on our health if remaining to be unfulfilled, definitely in times of increased stress (Sippel et al., 2015). Consequently, the fifth research question was whether posttrauma social support can help increase trauma resilience and thus diminish the impact on well-being. We hypothesize that social support can help people to overcome a traumatic event and reduce its harmful effects on psychological well-being.

Summarized, the procedure and our predictions were as follows. In a first memory coherence task, all participants will be asked to write for 10 min about a turning point memory. Then, the Maastricht Acute Stress Test (Smeets et al., 2012) will take place, here used as a lab analog of a traumatic event. This is a validated task consisting of the cold pressor test, combined with a mental arithmetic task and social evaluation, known to evoke a sharp increase in acute stress. The MAST is not a mere stressor that takes place in the outside environment, but something that the participant needs to partake in him/herself (e.g., having to do the arithmetic task, having your own face videotaped, receiving personal feedback on your own presentation by the experimenter). Thereby, the MAST has a stronger relation to the self, in comparison with usual traumatic procedures (e.g., videos are merely watched but not experienced), which is why it was especially suited to use as a lab analog of a real autobiographical traumatic experience, which is also something that the individual experiences him/herself. Namely, we know from research that especially the coherence of those events that are highly identity relevant is related to outcomes of well-being (Waters and Fivush, 2015). Then, the experimental manipulation will take place, in which half of the participants will be socially supported by the experimenter, whereas the other half will not be. The second measurement of memory coherence will happen thereafter, during which participants will be asked to write about their experience of the traumatic event for 10 min. Before and after every step in the procedure, state anxiety and cortisol levels will be measured. We expect the coherence of the turning point memory to be higher than the coherence of the traumatic event. We also predict that coherent narratives will relate positively to lower state anxiety scores in comparison with incoherent narratives. Furthermore, we predict that coherence of the turning point memory can protect against the impact of the traumatic event on state anxiety. Lastly, we expect that social support after the traumatic event will have beneficial effects on the coherence of the traumatic narrative as well as on state anxiety afterwards.

## Methods

### Participants

A total of 50 adults took part in this experiment, after signing up for the study *via* the online Experiment Management System (https://psykuleuven.sona-systems.com/Default.aspx?ReturnUrl=%2f) of the university. Prior to sign-up, participants were informed of the exclusion criteria that were of relevance for this study. Since we are administering a stressful event and measuring cortisol in the procedure, a rather extensive exclusion criteria list, based on Smeets et al. (2012), was used. It consisted of cardiovascular diseases, hypertension, psychopathology (current or in the past), substance abuse, smoking >10 cigarettes/day, physical illness (for example, fibromyalgia), or medication that affects the HPA axis. Furthermore, participants needed to have Dutch as their mother tongue. Beside these exclusion criteria, only females who used the contraceptive pill and were not in their stop week during the experiment were allowed to participate, to prevent hormonal fluctuations that might affect cortisol measures. Moreover, there were strict rules of participation, including not drinking alcohol or doing any strenuous physical exercise for 24 h before participation and no teeth brushing, drinking, or eating 2 h before. Participants were sent a reminder *via* e-mail of all these rules 1 day before participation. During the experiment, five participants decided to stop due to pain, feeling unwell or uncomfortable. Those five students have been excluded from the further data analysis. The final sample consisted of 45 adults between the ages of 18 and 29, *M* = 20.98, SD = 2.32, of which 35 (77.8%) were female and 10 (22.2%) were male. All participants gave written informed consent before the start of the study and received either course credit or remuneration (€8) for their participation. The study was approved by the KU Leuven Social and Societal Ethics Committee (G-2018 11 1395).

### Materials and Measures

#### Memory Coherence

Memory coherence was measured twice during the study, the first time about a turning point memory and the second time about their memory of the traumatic procedure in the experiment itself. To recall the turning point memory, participants were asked to think back over their life and identify an event that changed their life or the kind of person they are and that they now see as a turning point. They could choose something from any area of their life (relationships, work, other interests) and were asked to describe what happened, when it happened, who was involved, what they were thinking and feeling, why this experience was significant, and how it changed their life or them as a person. They had to think and write about this for a minimum of 10 min before they could go on to the next step of the experiment. For the second narrative, they received similar instructions but adapted to the recall of the traumatic task. Participants were asked to think back of the task did or just did in the experiment and were asked to describe what happened, when it happened, who was involved, and what they were thinking and feeling. Again, they were given a minimum of 10 min to do so. All memories were manually coded—blind for condition—according to the Narrative Coherence Coding Scheme (NCCS; Reese et al., 2011). Using this coding scheme, each narrative was assigned a total score from 0 to 9, consisting of the sum of the scores on the three dimensions that the scheme entails, namely context (0–3), chronology (0–3), and theme (0–3). To make sure the data were coded reliably, two raters independently coded all narratives and used a consensus system afterwards to determine the final score.

#### Traumatic Event

As an experimental analog for a traumatic event, while still adhering to ethical norms, we chose to use a task that is known to induce a strong and acute stress response. The Maastricht Acute Stress Test (MAST, Smeets et al., 2012) is a protocol that combines physical, cognitive, and psychosocial stress and has proven to elicit subjective and neuroendocrine stress responses (Quaedflieg et al., 2017; Shilton et al., 2017). It can be considered a lab analog of a traumatic event, since both the MAST and real-life traumatic experiences have shown to evoke powerful measurable stress responses (Desborough, 2000; Smeets et al., 2012). For the execution of the MAST, we used the same exact procedure and material as Smeets et al. (2012) did. The MAST consisted of a 5-min preparation phase in which the participant is informed about the different parts of the task *via* a PowerPoint presentation in combination with instructions read out loud by the experimenter. Participants received the information that they would be asked to immerse their hand in an ice bath for multiple trials, of which the duration would be randomly chosen by the computer, yet never take longer than 90 s. In reality, the duration of all trials was preset by the computer and fixed for all participants. The acute stress phase took place after the preparation phase and lasted for 10 min. It involved alternation between a cold pressor test (CPT) and a mental arithmetic task, combined with social evaluation aspects of videotaping and feedback from the experimenter. In between the trials, participants could lay their hand on a towel and had to engage in the mental arithmetic task, which they were told would take at least 45 s. The arithmetic task involved counting backwards from 2,043 in steps of 17, during which the experimenter encouraged them to do this as accurate and fast as possible and provided negative feedback when mistakes were made, upon which they had to start over at 2,043. They were asked to look in the camera during the entire process, as their facial expressions would be videotaped to be later analyzed by the experimenter. On the last PowerPoint slide of the MAST, participants got the message that they were going to have a rest period between trials. However, without participants knowing, this was the end of the MAST.

#### Social Support

The manipulation of this study concerned the social support given to the participant after the MAST by the experimenter. In the experimental condition, the experimenter supported the participant and an empathic conversation was held about the participant's traumatic experience. In the control condition, the experimenter gave negative feedback about the performance without expressing empathy for the subjective stress of the participant. In both conditions, every element of the stress protocol (COPT, arithmetic task, videotaping) was discussed, but the crucial difference was the way in which the experimenter expressed empathy and gave positive feedback on the participant's experience vs. was not empathetic and gave negative feedback concerning the participant's performance.

#### Cortisol

Salivary cortisol was assessed as a measure of a neuroendocrine stress response (HPA axis). Saliva samples were collected using synthetic Salivette (Sarstedt®) devices at five different times during the experiment. Samples were stored in a freezer at −18°C on the day of collection.

#### Psychological Well-Being

For our measure of momentary psychological well-being, we chose to work with State Anxiety questionnaires (STAI-S; Spielberger et al., 1983). They are a particularly relevant measure in order to measure well-being after trauma, since anxiety disorders (not only PTSD, but also GAD and PD) are the main risk to our psychological well-being after trauma exposure (Ayazi et al., 2014). Furthermore, STAI-Ss are able to capture moment-by-moment fluctuations in anxiety, by administering them five times throughout the experiment. We also administered the Trait Anxiety Inventory (STAI-T; Spielberger et al., 1983) in the beginning of the experiment to be able to control for group differences in base level of anxiety. The STAI-S and STAI-T questionnaires have 20 items to be rated on a 4-point scale, with state instructions assessing how anxious people feel at that particular moment and trait instructions assessing anxiety in general, across situations. Internal consistency for the STAI is good (0.86 ≤ Cronbach's α ≤ 0.95), and test-retest reliability has also proven to be more than sufficient (0.69 ≤ *r* ≤ 0.89) (Spielberger et al., 1983).

### Procedure

Participants were tested individually and after 1 pm, to ensure a stabilized endocrine response. Upon entering the lab, the rules for participation were checked. And if participants had adhered, they were sat down in a room behind a computer screen. After filling out the informed consent, participants were randomly assigned to the experimental or control condition, which remained blind to them. An overview of the study design and precise timings is presented in Table 1. A saliva sample was collected 15 min after the participant arrived, to measure baseline cortisol. Subsequently, the STAI-T and the first STAI-S were administered. Then, the first memory coherence measure took place, in which participants wrote for 10 min about a turning point memory. Hereafter, objective stress (cortisol) and subjective anxiety (STAI-S) measures were taken. Then, the MAST took place, which consisted of a 5-min preparation phase and a 10-min acute stress phase. After the MAST, which was concealed to participants as a rest period between test trials, cortisol and STAI-S measures were taken again. Hereafter, participants were told the procedure was over and no further trials would follow. Then, the experimental manipulation took place, in which participants were socially supported or not supported. Subsequently, we measured cortisol and state anxiety again. The second measurement of memory coherence happened thereafter, during which participants wrote about their experience of the traumatic procedure for 10 min. Finally, cortisol and STAI-S measurement were taken once more as a follow-up measure. At the end of the experiment, participants were debriefed about the goals of the study, the experimental manipulation and any other questions they had were answered. The main research questions, variables, conditions, and analyses were preregistered on AsPredicted http://aspredicted.org/blind.php?x=7s32jw.

**Table 1:** Study timings and design.

**Time (min)**	**Task**
0	Start-up, informed consent
1	CORT 1
3	STAI-T, STAI-S1
5	MC1 (turning point)
15	CORT 2
17	STAI-S2
19	MAST
31	CORT 3
33	STAI-S3
35	SS or no SS
40	CORT 4
42	STAI-S4
44	MC 2 (traumatic event)
54	CORT 5
56	STAI-S5
58	Debriefing

*CORT, cortisol; STAI-T, STAI-S, trait and state anxiety; MC, memory coherence; MAST, Maastricht Acute Stress Test; SS, social support; no SS, no social support*.

## Results

### Data Analysis

Data were analyzed with IBM SPSS Statistics 25, using an α-level of 0.05 for all analyses. We investigated our research questions and according hypotheses using *t*-tests, repeated measures ANOVAs, Pearson correlations, and linear regressions. We conducted a *post-hoc* power analysis using G^*^Power (Faul et al., 2007). Based on the mean observed *R* square (*R*^2^ = 0.21) as an effect size estimate and a critical alpha of 0.05, we reached a very good power of 0.93, which showed that our sample (*N* = 45) was sufficiently large to detect significant associations.

### Coherence of Traumatic Narratives

In order to investigate the coherence of traumatic and non-traumatic memories, paired-sample *t*-tests were used. We predicted that the coherence of traumatic narratives would be lower than the coherence of the non-traumatic turning point memories. Descriptive statistics of memory coherence for the turning point memories (MC1) and the traumatic memories (MC2) are presented in Table 2. In line with our hypotheses, the coherence of traumatic memories was significantly lower than the coherence of turning point memories, *t*(44) = 5.85, *p* < 0.001. Looking at the individual dimensions of memory coherence, data showed that both context, *t*(44) = 8.63, *p* < 0.001, and theme, *t*(44) = 4.62, *p* < 0.001, were lower for traumatic memories, whereas chronology was higher, *t*(44) = −2.66, *p* = 0.01.

**Table 2:** Descriptive statistics.

**Variables**	**Measure**	**Social support**		**No social support**	
		**M**	**SD**	**M**	**SD**
Coherence					
Total coherence	1	6.67	2.20	6.43	1.63
	2	5.13	1.57	4.67	1.59
Context	1	1.92	1.10	1.52	0.87
	2	0.29	0.69	0.24	0.70
Chronology	1	2.17	0.96	2.19	0.87
	2	2.75	0.68	2.38	0.92
Theme	1	2.58	0.72	2.71	0.46
	2	2.08	0.97	2.05	0.87
STAI-State	1	33.88	6.40	36.38	10.61
	2	33.50	6.72	34.48	10.79
	3	45.79	10.92	46.86	11.90
	4	35.04	9.43	37.71	10.33
	5	31.88	7.36	33.33	9.35
STAI-Trait		34.46	7.35	42.62	11.58
Cortisol	1	5.25	3.03	7.31	7.27
	2	5.49	2.88	6.71	6.35
	3	6.77	2.94	7.38	5.70
	4	10.50	5.50	9.26	6.56
	5	13.75	8.35	11.71	11.17

### Relations Between Coherence and Well-Being

To investigate our second research question, which involved the relations between coherence and well-being, we used correlations and regression analyses. We predicted that coherent narratives would reflect higher well-being (i.e., lower anxiety), in comparison with incoherent narratives. Correlations between memory coherence of the MC1, the MC2, and well-being (state and trait anxiety) are presented in Table 3. Thematic coherence did relate to anxiety levels after the manipulation and at follow-up; however, patterns were in the opposite of the expected direction. Individuals who were more thematically coherent in their turning point memory showed more symptoms of state anxiety later on. Furthermore, state anxiety related to coherence of the traumatic event as well. Follow-up regression analyses showed that participants who felt more anxious, right before the MAST, *β* = 0.41, *t* = 2.91, *p* = 0.006, at the peak, *β* = 0.35, *t* = 2.48, *p* = 0.017, and right after the MAST, *β* = 0.31, *t* = 2.09, *p* = 0.043, showed higher levels of thematic coherence afterwards. Possible explanations of these findings will be discussed below.

**Table 3:** Correlations between thematic (THE) and total memory coherence (MC) for turning point memories (THE1, MC1), traumatic memories (THE2, MC2), and anxiety levels (STAI).

		**THE1**	**MC1**	**THE2**	**MC2**
STAI-S1	*r*	0.19	0.12	0.24	0.28
	*p*	0.20	0.44	0.11	0.06
STAI-S2	*r*	0.29	0.27	0.41^*^	0.45^*^
	*p*	0.05	0.08	**0.01**	**0.00**
STAI-S3	*r*	0.29	0.15	0.35^*^	0.24
	*p*	0.05	0.31	**0.02**	0.11
STAI-S4	*r*	0.34^*^	0.27	0.30^*^	0.22
	*p*	**0.02**	0.08	**0.04**	0.14
STAI-S5	*r*	0.33^*^	0.19	0.23	0.18
	*p*	**0.03**	0.22	0.14	0.23
STAI-T	*r*	0.21	0.14	0.23	0.08
	*p*	0.16	0.37	0.13	0.62

* *Bold indicates significance at the 0.05-level*.

### Buffering Effect of Coherence Against the Impact of Trauma on Well-Being

To test if baseline narrative coherence could protect against the effect of trauma on well-being, we ran regression analyses. Contrary to our expectations, narrative coherence at baseline could not buffer against the impact of trauma on well-being. Regression analyses showed that coherence of the turning point memory could not predict the relative increase in state anxiety levels from right before to the peak of the MAST (STAI-S3–STAI-S2), *β* = −0.07, *t* = −0.47, *p* = 0.64. Neither was there a protective effect of narrative coherence when social support was added to the model, *β* = −0.001, *t* = −0.007, *p* = 0.99.

### Effect of Social Support on Coherence

In a fourth research question, we asked whether social support could affect coping with trauma, as reflected in the coherence of traumatic narratives. This question was examined using independent sample *t*-tests and repeated measures ANOVAs. We predicted that participants who would receive social support after experiencing the traumatic event, would write more coherently about it afterwards, in comparison with those who did not receive social support. However, results showed that support or no support after the traumatic event, did not significantly impact memory coherence of the traumatic event (MC2), as there was no main effect of condition, *t*(43) = 0.97, *p* = 0.34 on MC2. None of the individual dimensions differed between conditions either, as is shown for context, *t*(43) = 0.26, *p* = 0.80, chronology, *t*(43) = 1.55, *p* = 0.13, and theme, *t*(43) = 0.13, *p* = 0.90. Neither was the decrease in memory coherence from MC1 to MC2 impacted by the level of social support, as there was no interaction between total coherence and condition, *F*(1, 43) = 0.15, *p* = 0.70, nor between contextual coherence and condition, *F*(1, 43) = 0.99, *p* = 0.33, between chronological coherence and condition, *F*(1, 43) = 1.73, *p* = 0.20, or between thematic coherence and condition, *F*(1, 43) = 0.43, *p* = 0.52. In this study, the given support could thus not help to prevent a decrease in memory coherence after the traumatic event.

Memory coherence of the turning point and the traumatic event were related, *r* = 0.44, *p* < 0.001, but this relation was not better predicted by including the impact of the traumatic event (STAI-S3) or the impact of social support on anxiety levels (STAI-S4), as is shown in Table 4. The combination of MC1 and peak state anxiety (STAI-S3) could not predict MC2 better than MC1 alone could. Neither could the combination of MC1 and anxiety right after the support manipulation (STAI-S4) predict MC2 better, than MC1 alone could.

**Table 4:** Prediction of MC2 based on MC1, peak anxiety (STAI-S3), and anxiety after support (STAI-S4).

**Model**		***R^**2**^***	***B***	***SE***	**β**	***t***	***p***
1		0.19					
	(Constant)		2.56	0.76		3.36	<0.001
	MC1		0.36	0.11	0.44	3.21	<0.001
2		0.23					
	(Constant)		1.55	1.09		1.43	0.16
	MC1		0.34	0.11	0.41	3.00	0.01
	STAI_S_3		0.03	0.02	0.18	1.30	0.20
3		0.21					
	(Constant)		2.05	0.99		2.07	0.04
	MC1		0.33	0.12	0.41	2.87	0.01
	STAI_S _4		0.02	0.02	0.12	0.81	0.42

*Dependent variable: MC2*.

### Effect of Social Support on Well-Being

**Figure 1:**
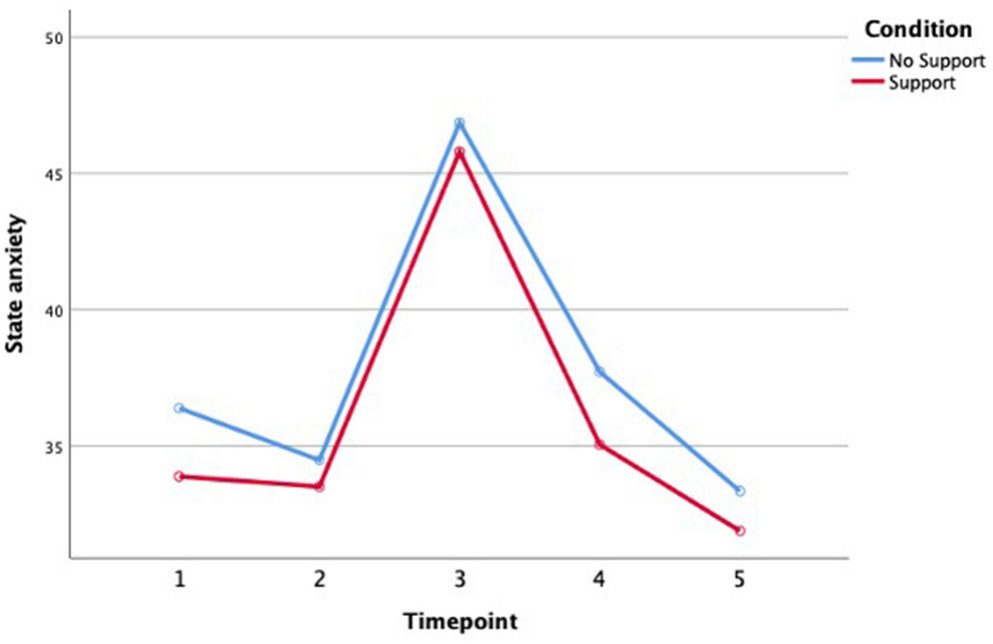
State anxiety over time for both conditions.

Fifth, we investigated the effect of social support on well-being using independent sample *t*-tests and repeated measures ANOVAs. We expected that social support posttrauma would help participants to feel better, and thus score lower on state anxiety measures after the traumatic event. State anxiety increased significantly from before to the peak of the traumatic procedure, *F*(1, 43) = 98.10, *p* < 0.001, and decreased again significantly afterwards, *F*(1, 43) = 74.10, *p* < 0.001. However, the support manipulation did not cause any differences in state anxiety after the MAST (STAI-S4), *t*(43) = 0.91, *p* = 0.37, or at follow-up (STAI-S5), *t*(43) = 0.56, *p* = 0.56. Neither were decreases afterwards moderated by social support, as there was no interaction between state anxiety and condition from the peak to right after the MAST (from STAI-S3 to STAI-S4), *F*(1, 43) = 0.47, *p* = 0.50 (see Figure 1).

### Exploratory Analyses

We investigated not only psychological but physiological stress responses as well, using cortisol measures, as a manipulation check for the trauma induction and to exploratory investigate it as a possible correlate of narrative coherence and state anxiety. However, the cortisol reactions of participants in this study were not in line with those usually found in the MAST procedure (Smeets et al., 2012).

**Figure 2:**
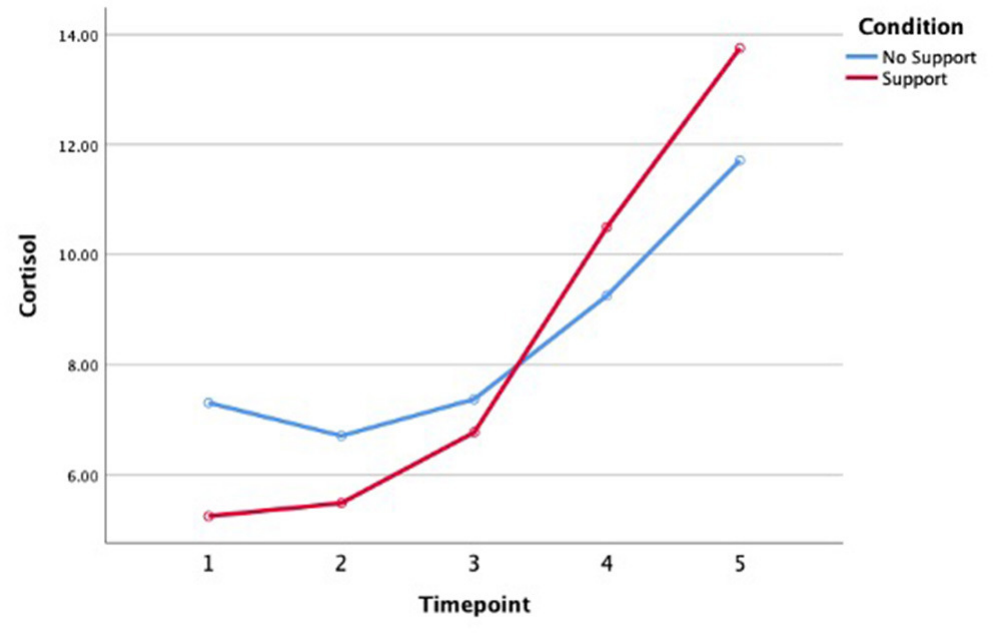
Cortisol over time for both conditions.

The MAST did cause an endocrinological stress response, measured *via* cortisol (see Figure 2). However, we saw an ongoing increase of cortisol from the beginning till the end of the experiment, which is not in line with previous studies using the exact same procedure (Smeets et al., 2012), in which cortisol usually peaks and then decreases again fairly soon afterwards. Furthermore, Figure 2 shows that the support vs. no support conditions did not impact the cortisol levels significantly.

Remarkably, regression analyses showed that cortisol levels at baseline were significantly negatively associated with memory coherence of the traumatic event. More specifically, participants who showed lower physiological stress levels at the first cortisol measurement right in the beginning of the study, were more coherent in their writing about the traumatic event, *β* = −0.30, *t* = −2.05, *p* = 0.047. Furthermore, participants who were still showing low physiological stress after writing about their turning point memory, were more coherent in the second writing task, *β* = −0.34, *t* = −2.39, *p* = 0.021.

## Discussion

The first research question concerned whether the coherence of memories of traumatic events was lower than coherence of turning point events. In line with our hypothesis, narrative coherence of the traumatic memory was poorer than coherence of the turning point memory. With regard to the individual dimensions of coherence, we saw that both context and theme were lower for traumatic memories. Only chronology was higher, which could be due to the specific kind of procedure that was used. The MAST is very stepwise in nature, which could evoke a strong chronological order in its description afterwards. Furthermore, the traumatic event took place more recently in the participant's life story than the turning point event. Therefore, it could be possible that, at least thematically, the traumatic event was not sufficiently processed yet, rendering an incoherent memory. This finding is in accordance with recent work suggesting that coherence in narratives is formed as a combination of both developmental as well as processing time (Fivush et al., 2017). Furthermore, turning point memories symbolize an important transition in life and are often practiced a lot more, mentally and socially, possibly enabling a more coherent narration than the new, traumatic experience in the lab that still needed to be processed (McLean et al., 2017). The stressful nature of the traumatic memory could also have taxed working memory more, thereby possibly hindering the adequate cognitive-emotional processing of events and rendering meaning-making more difficult, resulting in a lower score on narrative coherence (Klein and Boals, 2001).

However, memory coherence of the turning point event and the traumatic event were significantly correlated, *r* = 0.44, *p* < 0.001, which shows that both tasks do measure a similar individual difference variable, that can be flexible according to circumstances. Individuals seem to have a certain narrative style, which is under influences of the specific event (e.g., event type: Banks and Salmon, 2013) and the narrative context (e.g., responses of listener: Bavelas et al., 2000), which is in keeping with recent findings of Waters et al. (2019).

*Second*, we predicted that narrative coherence would relate positively to well-being or in our operationalizations relate negatively to state anxiety levels. Results were not in line with our predictions, as coherence did relate positively to anxiety levels during the experiment. Individuals who were more thematically coherent in their turning point memory showed more state anxiety later on. Furthermore, being more anxious in the beginning of the experiment and during the traumatic procedure predicted higher (predominantly thematic) coherence in the trauma narratives afterwards. A possible explanation for these findings is that the thematic component of coherence captures, in some instances, a form of rumination or unfinished attempts in meaning-making, which reflect anxious feelings (Buxton, 2016; Vanderveren et al., 2020b). For instance, anxious participants could have been dwelling on how the traumatic event made them feel all sorts of negative emotions, which gave rise to a high score on the thematic component in the Narrative Coherence Coding Scheme (NaCCs, Reese et al., 2011), but which does not reflect adaptive coping. This is in line with research of Sales et al. (2013), who also found that particular types of narrative meaning-making may reflect continuous and unsuccessful efforts in a search for meaning and may hence be more similar to rumination than to resolution. They also indicated that having an external locus of control, or low self-efficacy, might be a moderator in the relationship between cognitive processing and well-being (Sales et al., 2013). For participants who feel in control of their surroundings, thinking about the past might be beneficial for processing events, whereas for participants who feel anxious and out of control, thinking about the past might quickly turn into maladaptive ruminative thinking and increase depressive symptoms. This is in line with a rather broad field of research on the relation between locus of control and resilience (e.g., Lazarus and Folkman, 1984; Mor and Winquist, 2002). Furthermore, as cortisol responses indicated, stress was constantly high during the experiment. These ongoing high stress levels could have taxed participants working memory, hindered event processing, leading participants to detrimentally ruminate instead of proficiently make meaning out of what happened (Klein and Boals, 2001). Future research could investigate particular subcomponents of the thematic narrative coherence component to help explain adaptive (resolution) and non-adaptive (rumination) emotional processing.

In a *third* research question, we asked whether higher narrative coherence at baseline could buffer against traumatic events later on and thus indicate higher traumatic resilience. Results did not confirm our prediction, as coherence could not predict the increase in anxiety levels from before to the peak of the traumatic event. Although contrary to our predictions and some literature (Mason et al., 2019; Vanderveren et al., 2020a), there is another line of literature that indeed suggests that prior psychological adjustment is one of the smaller predictors of PTSD symptoms after trauma exposure (Ozer et al., 2003). Future research could further investigate possible buffering effects, since they could be a major point for primary prevention, thereby protecting people before harmful events take place.

*Fourth*, we investigated the impact of social support on narrative coherence. We did not find evidence that social support of the listener positively impacted memory coherence of the speaker, which is inconsistent with previous studies that have repeatedly shown that unfamiliar listeners do have an effect on the way we talk about our memories and remember past experiences (e.g., Bavelas et al., 2000; Pasupathi and Rich, 2005; Pasupathi and Billitteri, 2015). Possibly, the lack of an interactive dialog may have contributed to this effect. Participants wrote a narrative about the traumatic experience, after they had received supportive or unsupportive feedback regarding their performance on the task, so there was no real co-construction or collaboration in the process of narrative shaping (Pasupathi, 2001). This would be in line with research showing that, in particular with regard to processing highly difficult experiences, social interaction with loved ones or a professional therapist is necessary in order to create a structured and emotionally regulated narrative (White and Epston, 1990; Fivush and Sales, 2006; Lely et al., 2019). This relates to another limitation of the study, namely that the participant was not familiar to the experimenter. Research shows that the source of social support (e.g., family, friends, a significant other) is important in impacting its effectiveness (Kugbey, 2015), which will be further addressed below.

In a *fifth* question, we investigated if social support could increase traumatic resilience and thus protect against the harmful impact of trauma on well-being. Contrary to a large body of research (e.g., Ozbay et al., 2007; Charuvastra and Cloitre, 2008), our findings did not confirm the beneficial effects of posttraumatic social support. Possibly, our findings could be influenced by limitations in the design. First, we did not include a separate manipulation check to control for the effectiveness of social support, apart from the psychological (STAI-S) and physiological (cortisol) stress measures. Looking at the cortisol responses, stress continued to increase during the whole experiment, which may be a sign that participants did not feel at ease and could have expected the stressful task to take place again later on in the experiment. This explanation would make sense keeping in mind that instructions in the MAST conceal the end of the experiment to be a short break, so participants could possibly be never fully relieved. Furthermore, the manipulation of social support may have been less credible or ineffective, given that both the traumatic event and the support was administered by one and the same person (the experimenter). On top of that, the experimenter changed roles from being a neutral to becoming either very empathetic or very apathetic, which may have increased overall distrust and stress. As indicated earlier, research shows that the effectiveness of social support is dependent on many factors, one of which is the source of social support (Barrera et al., 1981; Li et al., 2014; Nguyen et al., 2016; Alsubaie et al., 2019). Furthermore, research has indicated that unsupportive social interactions have been more strongly associated with trauma responses than supportive social interactions (e.g., Ullman and Filipas, 2001; Andrews et al., 2003; DeCou et al., 2017). Future research could take this into consideration when setting up experiments. For instance, comparing a condition in which participants can have a phone call to a (supportive) loved one after a traumatic event, to a condition in which they are only allowed to talk to an (unsupportive) experimenter, and to a control condition in which they could not talk to any other person, could help to disentangle this effect. In addition, while people who generally have high social support tend to have lower PTSD symptoms on any given day, average PTSD symptom severity does not seem to be associated with day-to-day fluctuations in the availability of social support (Dworkin et al., 2018). For the present study, this would imply that the social support right after the MAST task may have not been enough to buffer memory coherence or state anxiety, and rather that we would need a follow-up design to create a larger difference between conditions (more social support over time in supportive group, less social support over time in unsupportive group), in order to be able to pick up differences in posttraumatic responses, like state anxiety or memory coherence. This would also be in line with research suggesting that attentive listeners can assist in the coherent co-construction of autobiographical narratives over time, and with research suggesting that memories eventually become reconstructions of previous (social) narrations (Bavelas et al., 2000; Pasupathi, 2001; Pasupathi and Rich, 2005; Fivush, 2011). This would mean that multiple interactions with supportive, attentive listeners over time would be more helpful to protect memory coherence and mental health, in comparison with merely a single intervention of support right after the traumatic event.

Finally, our exploratory analyses indicated that psychological stress in the form of state anxiety and physiological stress in the form of cortisol did not run along similar patterns, contrary to our expectations and previous research (Smeets et al., 2012). However, we did find that lower cortisol levels at baseline and after the first writing task could predict higher coherence of the traumatic memory. Thus, it could be possible that those individuals who are better able to remain calm in situations that do arouse some people (e.g., participation in an experiment, thinking about important memories), are more likely to cope more adaptively with stressful situations later on. This could be explained by the fact that the ability to remain calm renders the individual with sufficient free working memory space, which is needed in order to process difficult events (Klein and Boals, 2001).

Besides the aforementioned limitations in the design, another limitation can be noticed. Our sample consisted mostly of young, female, white students and was thus very homogeneous. Participants were also excluded based on current or previous psychopathology, which to some extent reduces our ability to generalize findings to clinical samples including individuals experiencing PTSD and other stress-related psychopathology. Future research could take this into account by examining (sub-)clinical samples. Furthermore, it would be useful to assess participant's prior traumatic experiences and examine these in relation to coping abilities with new traumatic experiences, since there is some evidence showing the impact of prior trauma on how new traumatic events are experienced (Breslau et al., 2008; Schock et al., 2016).

Concluding, in this study, narratives of traumatic experiences were less coherent than narratives of turning point events. However, contrary to our predictions, coherence, and, in particular, thematic coherence, related positively to anxiety levels. This possibly reflects a non-adaptive component in thematic coherence that could be related to ruminative processes and unfinished attempts at meaning-making. Furthermore, coherence at baseline could not buffer against the impact of trauma on anxiety levels in this study. Contrary to our hypotheses, social support did not have the intended beneficial effects on coherence, neither on well-being. Multiple possible explanations are suggested. The source of support and the traumatic event was identical, namely the experimenter, who was unfamiliar to the participant as well as took on different roles over the experimental procedure, which likely reduced the effectiveness the credibility of the social support. Also, stress levels for all participants were constantly increasing over the procedure, as reflected by rising levels of cortisol. This could have overruled the effect of social support on coherence and well-being overall. Nonetheless, lower cortisol levels at baseline and after writing about the turning point memory predicted higher coherence in the trauma narratives. This may point out that the ability to remain calm in difficult situations does relate to the ability to cope adaptively with future difficult experiences. Further research on the relations between narrative coherence, social support, and trauma resilience is recommended.

## Data Availability Statement

The dataset generated for this study can be found in online repositories (Open Science Framework): doi: 10.17605/OSF.IO/3H7QM.

## Ethics Statement

The studies involving human participants were reviewed and approved by KU Leuven Social and Societal Ethics Committee (G-2018 11 1395). The patients/participants provided their written informed consent to participate in this study.

## Author Contributions

LV, TS, PB, and DH: conceptualization and writing (review and editing). LV: formal analysis, methodology, and writing (original draft). TS, PB, and DH: supervision. All authors contributed to the article and approved the submitted version.

## Conflict of Interest

The authors declare that the research was conducted in the absence of any commercial or financial relationships that could be construed as a potential conflict of interest.
